# A Dispersive Liquid–Liquid Micro–Extraction Technique for the Pre–concentration and Quantification of Vitamin D_3_ in Milk and Yogurt Samples Using a Non-Aqueous HPLC Method

**DOI:** 10.22037/ijpr.2019.1100634

**Published:** 2019

**Authors:** Maryam Ghalebi, Elnaz Tamizi, Shirin Ahmadi, Ahad Sheikhloo, Mahboob Nemati

**Affiliations:** a *Department of Pharmaceutical and Food Control, Faculty of Pharmacy, Tabriz University of Medical Sciences, Tabriz, Iran.*; b *Food and Drug Safety Research Center, Tabriz University of Medical Sciences, Tabriz, Iran. *; c *Pharmaceutical* *Analysis Research Center, Tabriz University of Medical Sciences, Tabriz, Iran.*

**Keywords:** DLLME, HPLC-UV, Vitamin D3, Milk, Yogurt

## Abstract

In present study, a DLLME-HPLC-UV method was developed and validated for the extraction, pre–concentration, and subsequently quantification of vitamin D_3_ (Vit D_3_) in milk and yogurt samples. In order to be able to extract Vit D_3_ from studied samples efficiently, the DLLME procedure was optimized with respect to the parameters affecting the extraction efficacy, where acetonitrile (2 mL as disperser solvent) resulting from the protein precipitation procedure was mixed with 80 µL carbon tetrachloride (as an extraction solvent) respectively. The extracted samples were quantitatively analyzed with a HPLC technique using a C_8 _column (250 mm × 4.6 mm, 5 μm) at room temperature (25 °C), mobile phase of acetonitrile/methanol (90:10% v/v) in isocratic elution mode at a flow rate of 1.2 mL/min and UV detection at 265 nm. The method validation results revealed that the method was linear in the concentration range of 2 to 60 ng/mL (r = 0.9997) with a LOD of 0.9 ng/mL and LLOQ of 2 ng/mL; the method was accurate (-2.1% ≤ RE% ≤ +0.6%) and precise (1.2% ≤RSD% ≤ 11.3%) and its recovery was in the range of 86.6 to 113.3%. The obtained results indicated that the method could be utilized as an easy to use technique for the monitoring Vit D_3_ in dairy products, especially milk and yogurt samples.

## Introduction

Cholecalciferol, vitamin D_3_ (Vit D_3_), is one of the isomers of vitamin D that is converted to its active form via hydroxylation in liver and kidney. It is of great importance in keeping normal calcium metabolism (1). In the other words, the main biological function of Vit D_3 _is to improve intestinal absorption of calcium and phosphate (2). 

The required Vit D_3 _for the human body can be provided through either exposure of its precursor (7-dehydrocholesterol), existing in the epidermal layer of skin, to ultraviolet (UV) radiation or food intake (3). However, it should be mentioned that the amount of Vit D_3_ in food resources is little and only fishery products, particularly fatty fish such as salmon, mackerel, and also, herring and fish liver oils, are the good sources of Vit D_3_ (4). In the case of dairy products, bovine milk is considered to be one of the Vit D_3_′s dietary origins, nevertheless the amount of Vit D_3_ in milk is too low and in the range of 4–40 IU/L (0.1–1.0 µg/L) (3). It has been demonstrated that Vit D_3_ deficiency is a common nutritional problem in all age groups in the world (5, 6). This deficiency could lead to the prevalence of rickets in children and osteomalacia in adults (7, 8). Owing to the important role of vitamin D_3 _in human health, the development of analytical methods applicable to determine the accurate amounts of this micronutrient in food stuffs is necessary (9).

Since vitamins are present in low quantities and in combination with other compounds in foodstuffs, different techniques such as saponification, liquid–liquid extraction (LLE), and solid-phase extraction (SPE) have been utilized for the cleanup and extraction of Vit D_3_ from food samples (10, 11). However, some drawbacks such as use of large volumes of toxic solvents and being time-consuming make these techniques as costly procedures which are incompatible with the environment.

In recent years, significant efforts have been devoted to achieve simple alternative methods for sample preparation. Dispersive liquid–liquid microextraction (DLLME) is a relatively new technique introduced by Rezaee *et al. *in 2006 (12). It is an easy to operate, rapid and environmentally friendly technique (13). Low amounts of required organic solvents and fast sample preparation procedure are the main advantages of DLLME (14).

Literature review revealed that different analytical methods mainly based on high performance liquid chromatography (HPLC) have been reported for determination of Vit D_3_ in foodstuffs (3, 7, 10 and 15-20). Analytical characteristics such as shorter analysis time, higher specificity and selectivity, acceptable precision and reasonable accuracy make HPLC as a favorable method for the food analysis compared to the other methods (21). An overview of the reported methodologies for the determination of Vit D_3 _in food samples was shown in Table 1. As it can be seen in this table, although DLLME technique was utilized for the extraction of Vit D_3_ from infant cereals, it has not been utilized for the extraction of Vit D_3_ in milk and dairy products so far (10). Therefore, since dairy products are accessible and affordable foods for different social classes that could be suitable carriers for Vit D_3 _fortification, the aim of the present study was optimization of a simple and sensitive DLLME technique coupled to HPLC with UV detection applicable to the quantification of vitamin D_3_ in some dairy products including milk and yogurt samples.

## Experimental

*Apparatus*

Analytical balance (A&D Weighing, San Jose, CA), vortex mixer (Scientific Industries Inc., Bohemia, NY), and high-speed centrifuge (Eppendorf, Hamburg, Germany) were used for sample preparation.

*Chemicals and Reagents*

*Reagents*

Chloroform, carbon tetrachloride, isopropyl alcohol, and ethanol were purchased from Merck (Darmstadt, Germany). Methanol and acetonitrile were obtained from Duksan Pure_Chemicals Co. (Gyeong gi-do, Korea). All solvents were of analytical or HPLC grade.

*Standard Solutions*

Cholecalciferol (Vit D_3_) was prepared from Sigma-Aldrich. Stock solution of Vit D_3_ with a concentration of 1000 μg/mL was freshly prepared via dissolving accurately weighed amount of cholecalciferol standard powder in chloroform. Working standard solutions of Vit D_3_, in a concentration range of 1 to 60 ng/mL, were prepared through diluting fresh stock solution using mobile phase (acetonitrile/methanol 90:10% v/v). All solutions were stored at 4 °C and covered with aluminum foil to be protected from light, prior to the analysis.

*HPLC-UV Instrumentation*

The chromatographic analyses were performed using a HPLC system (Knauer, Berlin, Germany). The detection was carried out using a UV detector (Knauer, Berlin, Germany) at 265 nm. The separation was achieved using a C_8 _column (250 mm × 4.6 mm, 5 μm) (Knauer, Berlin, Germany) at room temperature (25 °C). The mobile phase composed of a mixture of acetonitrile and methanol (90:10% v/v) used in isocratic elution mode at a flow rate of 1.2 mL/min.

*Sampling*

A total of 10 dairy products including 7 brands of milk and 3 brands of yogurt among available and well-known dairy brands in Tabriz, Iran were purchased from local supermarkets in a time period of May to July 2016. Among purchased products, four brands of milk samples (A, B, C, and E) and one brand of yogurt samples (D) were fortified with Vit D_3_. It should be mentioned that three samples of each brand with different batch numbers were prepared and analyzed in triplicate.

*Sample Preparation/Clean up Procedure*

One mL of milk samples and 1 mg of yogurt samples were shaken for 1min. Then, 2 mL of acetonitrile was added into the samples and vortex mixed for 2 min. After that, the samples were centrifuged for 10 min at 6000 rpm and finally the upper aqueous layer was transferred to another centrifuge tube for DLLME procedure.

*DLLME Procedure*

The applied DLLME technique was based on a previously reported work (10). However, some modifications were performed in reported technique to optimize the efficacy of the pre-concentration procedure for the intended purpose. In order to conduct DLLME, 80 μL of carbon tetrachloride as an extracting solvent was added to the obtained fraction from the previous step that was used as a dispersive solvent as well. After that the mixture was quickly injected into 5 mL of double distilled water using a syringe that resulted in the formation of a cloudy solution. Then, the mixture was gently shaken for 1 min followed by centrifugation at 11000 rpm for 2 min that led to the sedimentation of the extracting solvent at the bottom of the conical tube. Finally, the sedimented phase was totally transferred to a microtube and evaporated to dryness at room temperature. The residue was reconstituted with 50 μL of acetonitrile and 20 μL of the obtained solution was injected into the HPLC.

*Validation of Optimized DLLME-HPLC Method*

The optimized DLLME-HPLC technique was validated for the quantitative purposes based on the ICH guidelines on validation of analytical procedures (22). In order to investigate linearity of the method and lower limit of quantitation (LLOQ) and upper limit of quantitation (ULOQ) amounts, the peak areas of calibration samples with concentrations in the range of 2 to 60 ng/mL calculated from the equation of ″the obtained peak area from calibration sample – peak area of blank sample″ were plotted against Vit D_3 _concentrations (n = 3 at each concentration). For preparation of calibration samples, 100 µL of working standard solutions with concentrations in a range of 10–600 ng/mL were spiked into 1 mL of samples. To illustrate, 100 µL of a working standard solution with a concentration of 10 ng/mL was spiked into 1 mL of a milk sample to achieve a calibration sample with a concentration of 1 ng/mL.

LOD (limit of detection), of the method has been calculated using the equation of "LOD = 3.3 × σ/s", where σ is the standard deviation of peak areas from the analysis of six individual blank samples prepared based on the optimized conditions and s is the slope of the calibration equation. 

The precision, including repeatability and intermediate precision, and accuracy of the method were investigated at 3 concentrations at the lower, the middle, and the upper levels of the calibration curve (at 2, 20 and 60 ng/mL Vit D_3_). Repeatability and intermediate precision were derived from the calculated amounts for each concentration using the calibration equation and peak areas obtained from the repeated analyses (n = 3) in one day and 3 days in a row, respectively. For accuracy determination, the samples with known concentrations of 2, 20, and 60 ng/mL Vit D_3_ were analyzed in triplicate, then the experimentally derived concentrations were calculated from the peak areas and calibration equation. 

The accuracy of the method was presented as a relative error (RE%) calculated using the equation of ″((calculated concentration – nominal concentration)/nominal concentration) × 100″. The recovery of the method was evaluated at three concentration levels including 2, 10, and 40 ng/mL and reported as a percentage of the experimentally derived concentration to the nominal concentration.

## Results and Discussion

*Optimization of the HPLC Condition*

The chromatographic separation was optimized for the intended purpose. In order to select the stationary phase, performance of C_8 _and C_18 _(4.6 mm × 250 mm, 5 μm) columns for the separation of Vit D_3 _were investigated. Due to the poor peak shapes obtained using C_18 _column, C_8 _column was selected as the suitable stationary phase. To select the optimized mobile phase, different mixtures of solvent consisting of 5% acetonitrile in methanol, 10% acetonitrile in methanol, and 10% acetonitrile in isopropyl alcohol, were evaluated. 

**Figure 1:**
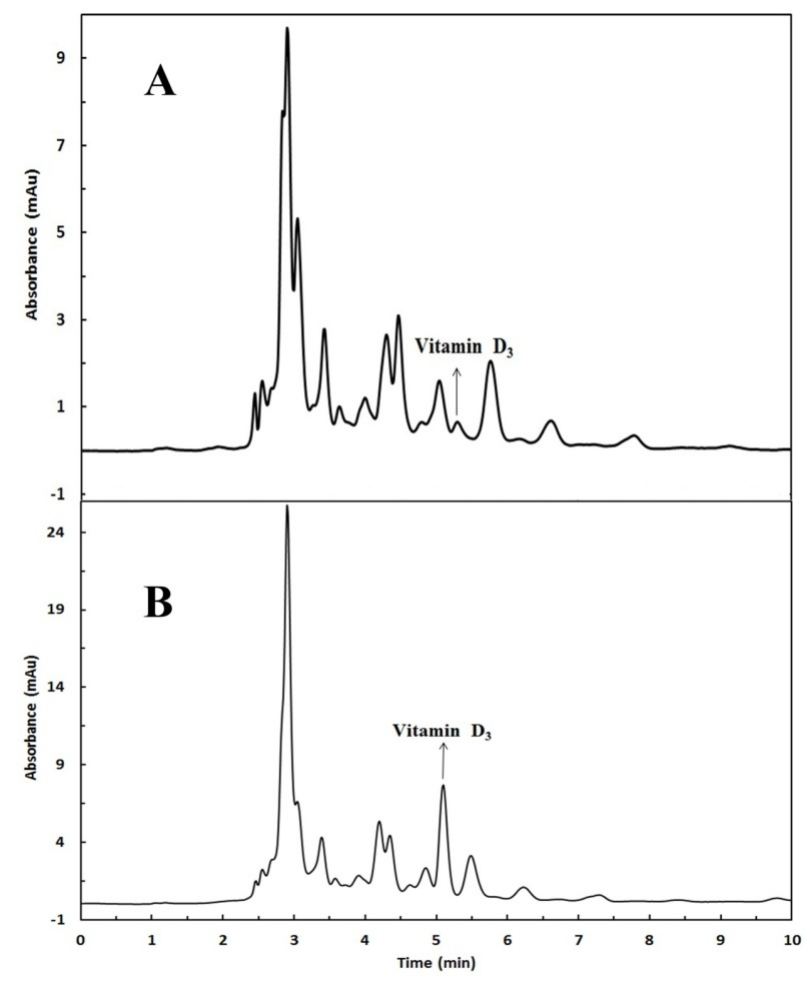
A dispersive liquid–liquid micro–extraction technique for the pre–concentration and quantification of vitamin D 3 in milk and yogurt samples using a non-aqueous HPLC method. The obtained chromatogram from the analysis of samples using a optimized HPLC method, separation conditions: C 8 column (250 mm × 4.6 mm, 5 μm), room temperature (25 °C), mobile phase of acetonitrile/methanol (90:10% v/v), flow rate of 1.2 mL/min, UV detection at 265 nm. (A) blank milk sample; (B) milk sample spiked with 50 ng/mL Vit D 3

The obtained results indicated that the best separation was achieved using the mixture of acetonitrile and methanol with a ratio of 90:10% v/v with flow rate of 1.2 mL/min and an isocratic elution mode at 25 ºC. The obtained chromatograms from the HPLC-UV analysis can be seen in Figure 1.

*Optimization of the DLLME Procedure*

*Selection of Extracting Solvent′s Type and Volume*

Selection of a suitable extracting solvent is a critical factor that affects the efficiency of the DLLME procedure. Low solubility in water, lower density than water, good chromatographic behavior and high affinity to the analyte of interest are important criteria to choose a suitable extracting solvent (23). In the present study, tetrachloroethane, carbon tetrachloride, and chloroform were utilized as extracting solvents. The extractions were carried out with injecting 80 μL of each extracting solvent in combination with 2 mL of acetonitrile extract (as a disperser solvent) into the 5 mL water. Considering the volume of sedimented phase and the required time for the evaporation of the applied solvent at the end of DLLME procedure, carbon tetrachloride was selected as the extracting solvent for the further analyses. In addition to the extracting solvent′s type, the impact of its volume on the efficacy of DLLME procedure was evaluated, where different volumes of carbon tetrachloride (80, 100, 150 and 200 μL) were utilized to accomplish the best extraction output. 

**Figure 2:**
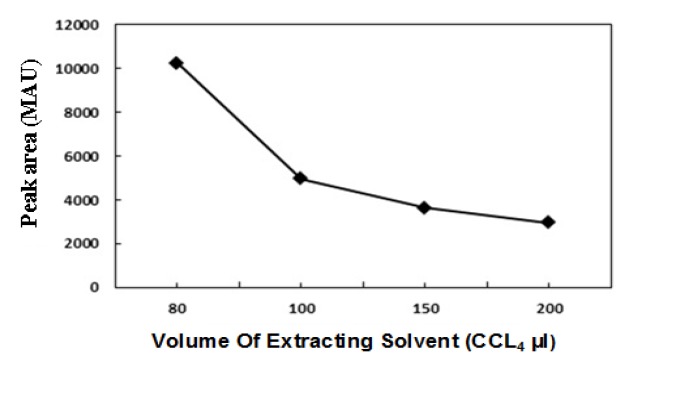
A dispersive liquid–liquid micro–extraction technique for the pre–concentration and quantification of vitamin D 3 in milk and yogurt samples using a non-aqueous HPLC method. Effect of extracting solvent′s volume on the obtained responses from the analysis of vitamin D 3 . Experimental conditions: type and volume of disperser solvent: acetonitrile, 2 mL; Vit D 3 concentration: 20 µg/mL; HPLC conditions: C 8 (4.6 mm × 250 mm, 5 μm) column, acetonitrile/methanol (90:10% v/v) with flow rate of 1.2 mL/min as a mobile phase, isocratic elution mode at 25 ºC, UV detection at 265 nm.

As shown in Figure 2, 80 μL carbon tetrachloride was selected as an optimal extracting solvent, since in low volumes of extracting solvent (<80 μL) the two phase system was not formed.

**Table 1:** The previously reported methods for the extraction and quantification of Vit D3 in foodstuffs

**Sample**	**Extraction procedure**	**Analytical method**	**Sample volume/weight**	**Extraction** **solvent's type and volume**		**LOQ** **(ng/mL)**	**Retention time (min)**	**Linear range (ng/mL)**	**Recovery**	**Reference**
Fresh bovine milk Commercial milk	SPE	LC–MS/MS	10 mL	Hexane 50 mL		0.1	17.7	-	30–40%	
Skimmed Milk Whole milk	Saponification	HPLC	15 mL	Diethyl ether in hexane 30 mL		2.6	16.0	20 – 1000	94–110%	
Infant cereals	DLLME	LC–DAD	0.2-2 g	Carbon tetrachloride 150 µL		1–100	12.6	-	95–103%	
Non-enriched milk Enriched milk	LLE	HPLC	100 mL	Hexane 100 mL		-	19.3	1 – 7.5	-	
Fortified infant formula Milk Milk powder	LLE	LC/MS	4 mL	Isooctane 10 mL		0.14	3.60	-	103.2%	
Whole milk	SPE	HPLC	100 g	Diethyl ether: petroleum ether		-	20.7	-	95.4%	
Milk	Saponification	LC-MS/MS	4 mL	Hexane: dichloromethane 12 mL		-	17.7	-	98.9%	
Fortified milk	Saponification - Extraction with hexane	UPLC– PDA	20 mL	Hexane 8 mL		-	11.9	2.5 – 60	93%	
Milk-based infant formulas	Non-heating saponification	LC-MS	0.5-2 g in 10 mL water	Isopropyl alcohol 10 mL		-	10.7	1 – 100	93–110%	
Milk and yogurt	DLLME	HPLC-UV	1 mL	Carbon tetrachloride 80 µL		2	5	2-60	86.7-113.3%	Present work

**Table 2:** The obtained results from the accuracy and precision evaluations

**Concentration (ng/mL)**	**Accuracy (RE%)**	**Repeatability (RSD%)**	**Intermediate precision (RSD%)**
2	-1.5	11.4	7.2
20	-2.1	7.9	4.4
60	+0.6	3.9	1.2

**Table 3:** The obtained results from the quantification of Vit D 3 in studied real samples using the developed DLLME-HPLC-UV method

**Real samples** **(milk and yoghurt brands)**	**Label claim (IU** * **/mL)**	**Vit D** **3 ** **(ng/mL) ± SD**	**Vit D (IU** * **/mL) ± SD** **3**
A	0.2	10.5 ± 1.5	0.42 ± 0.1
B	_**	12.4 ± 1.7	0.49 ± 0.1
C	0.2	17.1 ± 1.2	0.68 ± 0.1
D	0.5	14.3 ± 3.3	0.57 ± 0.0
E	0.1	6.30 ± 0.9	0.25 ± 0.5
F	_	9.10 ± 2.7	0.36 ± 0.1
G	_	7.60 ± 1.3	0.30 ± 0.0
H	_	6.40 ± 1.8	0.25 ± 0.1
I	_	5.20 ± 0.8	0.21 ± 0.0
J	_	6.30 ± 0.2	0.25 ± 0.0

* Each IU is equal to 25 ng Vit D_3_ .

** Not mentioned.

*Selection of Disperser Solvent′s Type and Volume*

Miscibility of disperser solvent with both the organic phase (extracting solvent) and the sample solution is an essential factor in the selection of a suitable disperser solvent. It helps to disperse the extracting solvent in aqueous phase and as a result improves the extraction performance (23). In this study, acetonitrile, ethanol, and methanol were utilized as disperser solvents. According to the obtained results, application of acetonitrile as a disperser solvent resulted in the acceptable extraction efficiency. In addition to the disperser solvent′s type, its volume plays an important role in the performance of extraction procedure, because it could directly influence formation of cloudy solution between ternary component solvent systems. Therefore, the impact of dispersive solvent’s volume on the extraction efficacy was evaluated, where distinctive volumes of acetonitrile (500, 1000, 2000 and 3000 μL) were utilized to conduct DLLME procedure. The observations revealed that at low volumes (>2000 μL), acetonitrile couldn′t appropriately scatter extraction solvent resulting in negligible formation of the cloudy solution. Additionally, it is worth saying that application of low volumes of acetonitrile could not effectively precipitate the proteins of the samples in the cleanup step. Higher volumes of acetonitrile (<2000 μL) resulted in increased solubility of analytes in the aqueous phase and consequently less extraction productivity. Therefore, acetonitrile with a volume of 2000 μL was selected as the optimal dispersive solvent.

*Selection of the Extraction Time*

In DLLME, extraction time is characterized as a time period between the injection of the blend of a disperser solvent, extracting solvent and sample in the water and centrifugation (23).

In the present study, observations showed that alterations in the extraction time from 30 sec to 2 min did not affect the extraction efficiency, significantly suggesting the independency of the applied DLLME technique from extraction time. However, in order to keep the uniformity among the different analyses, 1 min was selected as an extraction time.

*Method Validation Results*

Method validation results indicated that the method was linear in the concentration range of 2 to 60 ng/mL with a calibration equation of y = 452385x - 526/73, where y was peak area and x was concentration of Vit D_3_ in µg/mL, and correlation coefficient of 0.9997. The LLOQ and ULOQ of the method was 2 and 60 ng/mL, respectively, owing to the possibility of the quantifications with acceptable precision (RSD% ≤ 20%) and accuracy (|RE%| ≤ 20%) at mentioned concentrations.

Since the obtained standard deviation among the responses of 6 blank samples was 136.0, the LOD of the method was calculated as 0.9 ng/mL.

The accuracy and precision results are reported in Table 2. As it can be seen in this table, the method was accurate (-2.1% ≤ RE% ≤ +0.6%) and precise (RSD% of less than 11.4%) enough for the quantification of Vit D_3_ in the linear range, and its recovery was in the acceptable range of 86.7 ± 9.5% to 113.3 ± 5.6%. It should also be noted that the recovery of vitamin D_3_ in yoghurt samples (D, E and G brands), by the proposed method was from 88.55%, 89.23, and 99.05%, respectively.

*Real Sample Analysis*

The samples purchased from local supermarkets as described in sampling section, were analyzed using the optimized DLLME-HPLC-UV method. As it can be followed in Table 3, the method successfully quantified the amount of Vit D_3_ in different samples and the findings indicated that in only one brand of fortified milk samples the obtained amount of Vit D_3 _was in accordance with the label claim; the finding highlights the need to consider stricter legislation for the monitoring of the claims on the labels of dairy products in Iran.

## Conclusion

A DLLME-HPLC-UV method has been developed and validated for the reliable extraction and determination of Vit D_3_ in dairy products, particularly milk and yogurt. The inherent advantages of DLLME procedure including simplicity and time and cost effectiveness, low amounts of required organic solvents and smaller sample volume along with lower LOQ value and shorter retention time (5.0 ± 0.8 min) are the main superiorities of the developed method to the previously reported ones. Nevertheless, it is worth saying that application of more sensitive detectors such as mass spectroscopy would result in the lower LOD and LOQ values as it can be seen in Table 1. Finally, derived from the all above mentioned discussions, it can be concluded that the developed method could be considered as a promising technique in the monitoring of Vit D_3_ in dairy products and evaluation of the label claims of the fortified samples in food quality control laboratories. 
